# Mobilization of LINE-1 in irradiated mammary gland tissue may potentially contribute to low dose radiation-induced genomic instability

**DOI:** 10.18632/genesandcancer.50

**Published:** 2015-01

**Authors:** Lidia Luzhna, Yaroslav Ilnytskyy, Olga Kovalchuk

**Affiliations:** ^1^ Department of Biological Sciences, University of Lethbridge, Lethbridge, Canada

**Keywords:** ionizing radiation, LINE-1, mammary gland, c-myc

## Abstract

It is known that cellular stresses such as ionizing radiation activate LINE-1 (long interspersed nuclear element type 1, L1), but the molecular mechanisms of LINE-1 activation have not been fully elucidated. There is a possibility that DNA methylation changes induced by genotoxic stresses might contribute to LINE-1 activation in mammalian cells. L1 insertions usually cause major genomic rearrangements, such as deletions, transductions, the intrachromosomal homologous recombination between L1s, and the generation of pseudogenes, which could lead to genomic instability. The purpose of this study was to evaluate the effects of low and high doses of ionizing radiation on the DNA methylation status of LINE-1 transposable elements in rat mammary glands. Here we describe radiation-induced hypomethylation and activation of LINE-1 ORF1 in rat mammary gland tissues. We show that radiation exposure has also led to the translation of the LINE-1 element, whereby the 148 kDa LINE-1 protein level was increased 96 hours after treatment with a low dose and low energy level radiation and remained elevated for 24 weeks after treatment. The mobilization of LINE-1 in irradiated tissue may potentially contribute to genomic instability. The observed activation of mobile elements in response to radiation exposure is consistently discussed as a plausible mechanism of cancer etiology and development.

## INTRODUCTION

LINE-1 (long interspersed nuclear element type 1, L1) belongs to the family of non-long terminal repeat retrotransposons. With over 500, 000 copies, L1 comprises 17-18 % of the human genome and is capable of its own expansion and also mobilization of other non-L1 elements that may dramatically shape the genome [1, 2]. It is well known that most of the L1 elements are mutated, rearranged, and/or truncated (at the 5′ end) and therefore, are not capable of further retrotransposition. Nevertheless, a small subset (80-100) of the full-length L1 elements is active, functional and potentially capable of self-expansion [3, 4]. A retrotransposition-competent L1 element (RC-L1) is 6-7 kb in length and consists of 5′-untranslated region (5′-UTR) with its internal CpG-rich promoter, two non-overlapping open reading frames (1kb ORF1 and 3.8 kb ORF2) separated by a 63bp intergenic spacer, and a 206nt 3′-UTR terminator with a poly(A) tail [5]. The internal or “minimal promoter” is generally believed to be the most important region for successful transcription of L1. However, in their study Alexandrova and colleagues have shown that the promoter strength is mostly dependant on the 390-526 bp region within the human L1 5′-UTR. Deletion of this fragment resulted in the significant decrease of promoter activity [6]. In the same study the authors proposed a model in which an internal enhancer region (390-526) of L1 5′-UTR is responsible for recruitment of the transcription initiation complex and might serve as a basis for enhanceosome formation. This internal enhancer overlaps with the region of L1 5′-UTR that drives transcription in opposite direction suggesting the existence of bidirectional transcription of L1[6]. ORF1 encodes a 40 kDa (p40) protein with the RNA-binding activity, while ORF2 encodes a 150 kDa protein with N-terminal endonuclease and C-terminal reverse transcriptase enzymatic activities [7]. ORF1 proteins (ORF1p) are predominantly cytoplasmic and form large ribonucleoprotein (RNP) complexes with L1 RNA and DNA [8]. ORF1p is reported to possess chaperone activity and possibly be significant in reverse transcription reaction [9]. The function of ORF2p in retrotransposition is much well-determined, as the endonuclease nicks the target DNA strand exposing a 3′-hydroxyl group that primes reverse transcription of L1 RNA by reverse transcriptase. Such mechanism of retrotransposon replication is termed as target-site-primed reverse transcription (TPRT) [10].

L1 insertions usually cause major genomic rearrangements such as deletions, transductions, intrachromosomal homologous recombination between L1s, and the generation of pseudogenes that can lead to genomic instability [11, 12]. L1 transcription and integration into the genome leads to recombination events that harvest chimeric retrotranscripts or pseudogenes that consist of the fused DNA copies of various RNAs. One study reported at least 81 of such chimeric pseudogenes which were classified in nine families [13]. Usually these chimeric retrotranscripts are composed of the copies of transcripts of mRNAs, ribosomal RNAs, or snRNAs fused to the 3′ site of L1and of 5′ sites derived from nucleolar RNAs [14]. Most of RC-L1s are strongly methylated [15] or silenced by the RNA interfering pathway [16], but the loss of methylation or silencing can activate LINE-1, and such activity was shown to be linked to several diseases including cancers.

Several studies provide solid evidence that the insertion of LINE-1 into structural genes may play a role in the origin and/or progression of cancers. L1 retrotransposons were previously detected in significant amounts in breast cancer: in 7 out of 8 malignant cell lines and in 9 of 12 primary infiltrating ductal carcinomas [17]. Line-1 retrotransposons and ORF1p were also isolated and characterized in rat chloroleukemia cells [18]. Some of the early studies have shown L1 expression and ORF1p in human germ cell cancers (teratocarcinoma and choriocarcinoma cell lines) [19]. A very recent study demonstrated the up-regulation of LINE-1 together with another retrotransposon, SINE B1, at a very early stage of murine mammary tumorigenesis. Moreover, they reported that these retrotransposons were rapidly amplified during cancer progression [20]. Similarly, LINE-1 quantification in sera of breast cancer patients was shown to be useful for detecting early-stage breast cancer, and the copy number was correlated with tumor size [21]. The insertion of LINE-1 element into the c-Myc gene and the APC gene was shown in primary breast cancer and colorectal cancer, respectively [22, 23].

Epigenetic alterations are well known to cause gene expression changes and affect genome stability. The loss of DNA methylation is usually associated with gene activation, while hypermethylation silences genes. Both events are associated with cancer phenotype. Not surprisingly, the evidence of aberrant DNA methylation in LINE-1 retroelements exists and correlates with the activity of transposons. CpG-rich L1 promoter hypomethylation leads to the activation of ORF1 sense transcription in chronic myeloid leukemia (CML) and is associated with poorer prognosis for the cytogenic response to interferon and imatinib [24]. Furthermore, a decrease in methylation levels of L1 have been shown to be associated with breast cancer risk in a dose-dependent manner [25]. Similarly, the hypomethylation-induced activation of L1 has been reported in testicular tumor, prostate and hepatocellular carcinomas, and chronic lymphocytic leukemia [26-29].

Genomic instability that results from genetic and/or epigenetic changes and leads to carcinogenesis, is often associated with environmental factors. The one certain example is radiation-induced genomic instability (RIGI). The cytotoxic effect of ionizing radiation relies on the ability to damage DNA. Radiation induces a variety of DNA lesions including damage to nucleotide bases, cross-linking, DNA single- and double-strand breaks (Little, 2000). Ionizing radiation can also alter DNA methylation. In rodents, radiation exposure was shown to cause dose-dependent and sex- and tissue-specific global genome hypomethylation. When C57/BI mice were irradiated with X-rays in the dose range of 0.5-5 Gy, a dose-dependent loss of global methylation was detected in male spleen and female liver and spleen (Pogribny et al. 2004). Radiation-induced global hypomethylation in mice was correlated with a lower expression of both maintenance and *de novo* methyltransferases (Raiche et al. 2004). Similar results were found in the thymus of mice exposed to fractionated whole-body X-ray exposure. Global hypomethylation in the thymus was coupled with decreased levels of DNMT1, DNMT3a and b, methyl CpG binding protein 2(MeCP2) and methyl CpG binding domain protein 2 (MBD2) (Pogribny et al. 2005). DNA hypomethylation in mouse bone marrow was linked to radiation-induced leukemia (Giotopoulos et al. 2006). Similar molecular changes were found in the irradiated rat mammary tissues and were pronounced to cause genomic instability (Loree et al. 2006). When modulating global DNA hypomethylation in MCF-7/DOX cells with methylation agent SAM, cells were sensitized to radiation-induced apoptosis (Luzhna and Kovalchuk 2010). Ionizing radiation is also known to contribute to the mobilization of transposable elements. Retrotransposition of L1 was shown to be increased up to 4-fold in cultured cells subjected to gamma irradiation. The frequency of such retrotransposition was proportional to the level of phosphorylated H2AX foci [30]. Similarly, gamma irradiation induced a moderate increase in the Ty 1 element in *S. cerevisiae* [31]. L1 retrotransposition events were shown to regulate gene expression after 5 Gy of X-ray exposure in EA.hy926 LINE-1 cell clones [32].

All the evidence of the radiation-induced DNA methylation changes and LINE-1 retrotransposition allows suggesting a possible effect of ionizing radiation on methylation status and/or activation of L1. Here, we describe the radiation-induced hypomethylation and activation of LINE-1 ORF1 in the rat mammary gland. The mobilization of LINE-1 in the irradiated tissues potentially contributes to genomic instability and cancer initiation.

## RESULTS

The purpose of this study was to evaluate the effect of low and high doses of ionizing radiation on DNA methylation status of LINE-1 transposable element in the rat mammary gland. The animals received a whole body exposure of different combinations of radiation energy levels and doses: low energy levels and low doses of X-rays (30kVp, 0.1 Gy), high energy levels and low doses (80kVp, 0.1 Gy), high energy levels and intermediate doses (80kVp, 1 Gy), and high energy levels and high doses (80kVp and 2.5 Gy). We examined the role of DNA methylation in the activation of LINE-1 transposon following radiation exposure. An increased expression of retroelements may lead to genomic instability and cancer initiation in the breast tissue that is often exposed to radiation for diagnostic and therapeutic procedures.

### DNA methylation levels of LINE-1 ORF1 in the irradiated mammary gland

**Figure 1:**
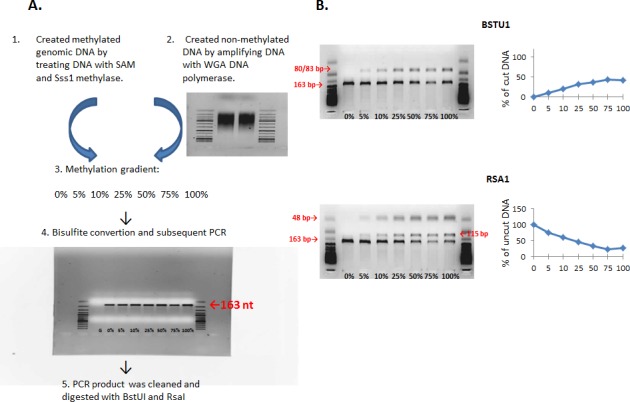
**A.** Preparation of methylation gradient for testing primers for the COBRA assay. Steps of preparation of methylation gradients (0% - 100% methylation) by mixing fully unmethylated and fully methylated DNA. **B.** A methylation standard after the digestion of bisulfite treated DNA with BstUI and RsaI restriction endonucleases. The higher the percentage of the cut 163-bp fragment by BstUI, the higher the methylation status of DNA. The higher the percentage of the uncut 163-bp fragment, the lower the methylation status of DNA.

**Figure 2:**
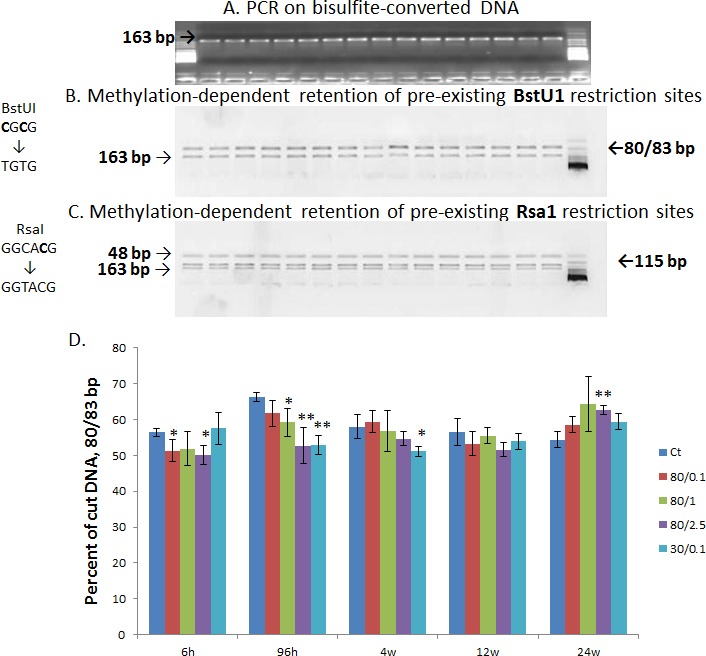
CpG methylation of LINE-1 promoter in the mammary gland of irradiated rats determined by the COBRA assay **A.** PCR amplification of the 163-bp from LINE-1 promoter. **B.** Methylation-dependent retention of pre-existing BstUI sites. Unmethylated CpG cytosines (highlighted) in the CGCG recognition sequence can be lost by bisulfite conversion, resulting in the uncut 163-bp fragments. Methylation at both sites allows the cleavage, resulting in 80/83-bp bands. **C.** Methylation-dependent retention of cytosine (highlighted) in the GGCACG sequence forms the RsaI recognition site, thus leading to the cleavage of the 163-bp fragment into 48- and 115-bp fragments. The loss of methylation at CpG cytosine sites will prevent the cleavage. **D.** Quantification of the cut BSTU1 fragments by AlphaView presented as mean values ± SD, n=4-6. * - significantly different from the respective control, p<0.05; ** - significantly different from the respective control, p<0.01, Student's t-test.

Methylation status of the LINE-1 regulatory region was determined by the COBRA assay [33]. This method is based on bisulfite modification of DNA - treatment of genomic DNA with bisulfite that converts unmethylated cytosines into uracils, while the methylated cytosines remain unchanged. The subsequent polymerase chain reaction (PCR) with primers corresponding to the regulatory region of rat LINE-1 results in the 163-nt fragment (Fig. 1, 2A). This fragment contains two sequences that are recognized by two endonucleases, BstUI and RsaI. BstUI digestion occurs at the recognition sequence CGCG only if both cytosines are methylated and thus protected from bisulfite conversion. Complete cleavage (in the case of complete methylation) results in two bands of 80 and 83 nt in length. Unmethylated DNA (converted) would resist cleavage and contribute to the163-nt band (Fig.1, 2B). An RsaI recognition site, GTAC, can be formed from the GGCACG sequence when non-CpG cytosine is unmethylated (therefore, converted), while CpG cytosine is methylated. Non-CpG cytosine methylation is very rare, and the RsaI recognition site is influenced mainly by the methylation status of CpG cytosine. Cleavage of the 163-nt fragment generates 48- and 115-nt bands, while the loss of CpG cytosine methylation prevents the cleavage and contributes to the 163-nt band (Fig. 1, 2C).

In order to ensure the validity of the assay and check the untested primers, a DNA methylation gradient and a methylation standard were prepared (0 - 100 % methylation), and PCR products were digested by BstUI and RsaI enzymes. Figure 1 represents the methylation standard curves and the sizes of digested fragments. Both BstUI and RsaI restriction reactions show methylation-dependent digestion: if unmethylated (0 % methylation), it resulted only in the 163-nt bands, whereas an increase in methylation led to the appearance of the 80/83-nt (for BstUI) and 115-nt (for RsaI) fragments (Fig. 1). The intensity of the 163-nt bands gradually decreased with an increase in DNA methylation. According to the methylation standard, the method is plausible and valuable in determining methylation levels of LINE-1 before and post radiation treatment.

The COBRA assay revealed hypomethylation of BstUI recognition sequences 96 hours after radiation treatment (Fig. 2). A significantly lower cleavage of PCR products digested by BstUI enzyme was observed in the case of intermediate and high doses at a high energy level (80kVp, 1 Gy and 80kVp, 2.5 Gy) and low doses at a low energy level (30kVp, 0.1 Gy). Hypomethylation was also observed at low doses with high energy exposure (80kVp, 0.1 Gy) at 6-hour time point, but it did not persist with time. Although such hypomethylation did not persist for longer than 4 weeks, it was indicative of immediate short-term LINE-1 reactivation which may contribute to genomic rearrangements and instability. Cytosine methylation at the RsaI site was not significant, and therefore data are not shown.

### LINE-1 ORF1 gene expression in the irradiated mammary gland

**Figure 3:**
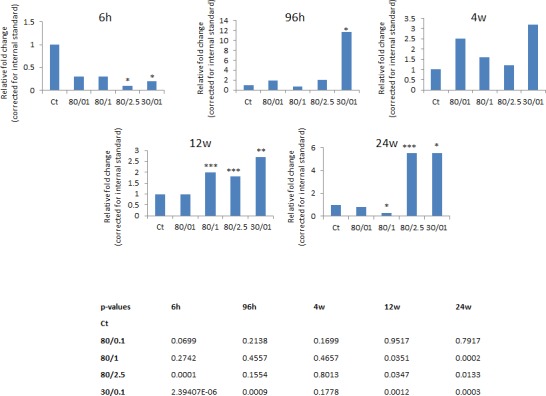
Fold change in the levels of Line-1 ORF1 transcript detected by qRT-PCR; Each treatment group was compared to its corresponding control B-actin was used as a reference gene (calculated by Pfaffl). * - significant, p<0.001; ** - significant, p<0.01; *** - significant, p<0.05 (Student's t-test).

The RT-PCR analysis was conducted in order to prove a hypothesis that the loss of DNA methylation may be correlated with the expression level of LINE-1. The expression of LINE-1 ORF1 in the irradiated mammary gland tissue was shown to be increased compared to controls (Fig. 3). Interestingly, initially (at 6 hours after exposure), the transcription level of ORF1 in the irradiated tissues was decreased in comparison to controls. Starting at 96 hours, the expression level returned back to the control point and was significantly elevated in the 30kVp/0.1 Gy treatment group. The high expression level of ORF1 was noticed 12 and 24 weeks after exposure in the 80kVp/1 and 2.5 Gy and 30kVp/0.1 Gy treatment groups (Fig. 3). These data suggest that the short-term hypomethylation of LINE-1 could possibly lead to a later more prolonged increase in LINE-1 ORF1 gene expression.

### LINE-1 and c-myc protein levels in the irradiated mammary gland

**Figure 4:**
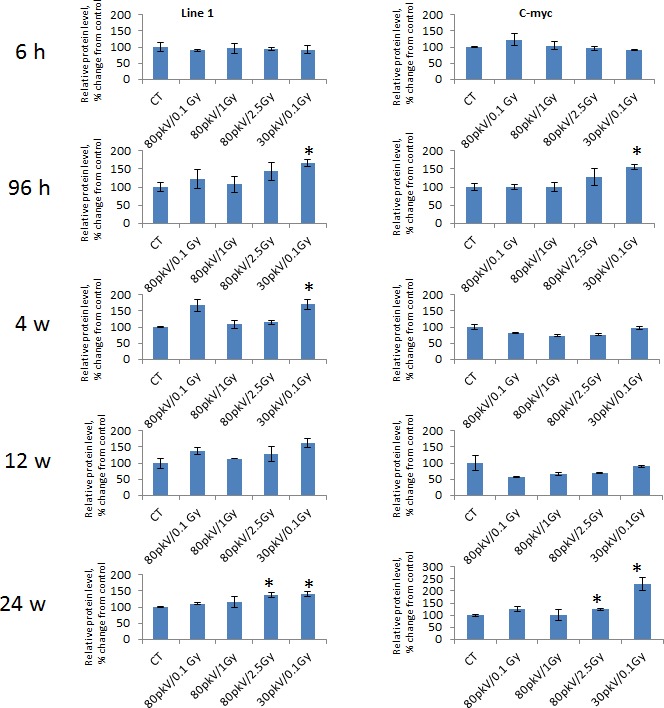
The levels of LINE-1 and c-MYC in the rat mammary gland upon whole body irradiation Protein levels relative to those in control non-irradiated animals are shown as Mean ± StEr. The representative blots from two independent experiments. * - p<0.05, student's t-test.

Having seen a pronounced and persistent LINE-1 gene expression, we proceeded with the detection of LINE-1 protein. A LINE-1 protein is an RNA-binding protein that has a high affinity to LINE-1 RNA, possesses endonuclease and reverse transcriptase activities, and forms ribonucleoprotein required for LINE-1 retrotransposition [5, 34-36]. We noted that the levels of LINE-1 protein were statistically significantly up-regulated 96 hours, 4 and 24 weeks after treatment with 30kVp and 0.1 Gy of X-ray and 24 weeks after treatment with 80kVp and 2.5 Gy of X-ray (Fig. 4, Suppl. Fig. 1). Such protein up-regulation is in agreement with RT-PCR results for the corresponding radiation doses and time points. Several studies demonstrated that hypomethylation-induced LINE-1 retrotransposition could activate certain oncogenes such as c-MYC [22]. We found that c-MYC protein expression was elevated 96 hours and 24 weeks after treatment with 30kVp and 0.1 Gy of X-ray and 24 weeks after treatment with 80kVp and 2.5 Gy of X-ray (Fig. 4, Suppl. Fig. 1). Undoubtedly, the elevated c-MYC protein level in the rat mammary gland tissue has resulted from radiation exposure and is possibly linked to LINE-1 hypomethylation and reactivation.

## DISCUSSION

A wide source of ionizing radiation exposure is delivered by medical diagnostic and therapeutic procedures [37]. Damage to DNA imposed by low and intermediate doses of ionizing radiation may initiate neoplastic development in a healthy mammary gland. The most critical types of damage caused by ionizing radiation are double-strand breaks (DSBs) in the DNA helix that can either force a damaged cell to programmed cell death (apoptosis) or can be repaired [38]. However, if repair mechanisms fail, cancer induction can start. There is a wide spectrum of mechanisms of radiation-induced cancer initiation. The activation of mobile elements in response to radiation exposure is consistently discussed as a plausible mechanism of cancer etiology and development. For instance, it has been shown that gamma radiation and chemotherapeutic drugs are associated with the induction of SINE expression [39]. Because of their mutagenic activity, transposons possess a threat to genome integrity [40]. LINE-1 expression is associated with DSB formation through L1-encoded endonuclease activity and may be a source of genotoxic stress in irradiated cells [30, 41]. Extrachromosomal accumulation of L1 DNA was reported in HIV-1 infected primary CD4(+) cells, while an increased retrotransposition of L1 was shown for HIV-1 infected Jurkat cells which could lead to HIV-1-induced genomic instability [42]. Line-1 endonuclease creates DSBs allowing new Line-1 copies to integrate into DNA [3]. Using tagged RC-L1 clones in cultured cells, it has been shown that approximately 10 % of LINE-1 insertions cause vast genomic deletions and chromosomal rearrangements that are a source of genomic instability [11, 12]. Although it has been known that cellular stresses such as ionizing radiation activate LINE-1, the molecular mechanisms of LINE-1 activation are not fully elucidated. There is a possibility that DNA methylation changes induced by genotoxic stresses might contribute to LINE-1 activation in mammalian cells. Stribinskis and Ramos discussed that the epigenetic dysregulation of retroelements in the BaP-treated cells may contribute to carcinogen-induced mutations and genomic instability [43]. Similarly, hypomethylation of LINE-1 has been reported in many cancers, and it has been suggested to promote genomic instability and facilitate tumor progression [24, 44].

Evidence presented in this report suggests that exposure to genotoxic ionizing radiation may involve the epigenetic activation of LINE-1 mobile elements in mammary tissue. The results of this study show the loss of CpG methylation in promoter region of LINE-1 in the rat mammary gland exposed to radiation. Interestingly, it was an early response (96 hours) to both high energy levels and intermediate-high doses and to low-energy levels and low doses. The methylation status of LINE-1 promoter returned to the control level and was not observed 12 and 24 weeks after treatment (Fig. 2). Several adaptive possibilities, such as changes in methyl group metabolism and the inactivation of DNA methyltransferases, could return the methylation status to its original level. Nevertheless, short-term hypomethylation was associated with a long-term reactivation of LINE-1. According to the qRT-PCR analysis, the high elevation of LINE-1 ORF1 gene expression was observed and persisted for 12 and 24 weeks, regardless the fact that the methylation level of LINE-1 was restored at these time points (Fig. 3). Such phenomenon is interesting on its own, but it is difficult to explain at the moment without further investigation. Considering that LINE-1 retrotransposon activation may lead to its migration and insertion into the genome, we assume that one or few copies could be inserted in a very active genome region and remain unmethylated there retaining a very high copying potential. To prove this suggestion, a further analysis of copying ability of LINE-1 has to be performed.

Radiation exposure has also led to the translation of LINE-1 element. The level of a 148-kDa LINE-1 protein was increased at 96 hours after treatment with low-energy-level and low dose radiation and remained high at 24 weeks after treatment (Fig. 4, Suppl. Fig. 1). Meanwhile, a significant elevation of LINE-1 protein was also detected at 24 weeks after treatment with high energy/high dose radiation. The LINE-1 protein possesses an endonuclease and reverse transcriptase activities [35, 36]. The ORF1 and ORF2 proteins associate with their encoding transcript, form a retrotransposition unit that allows for the retrotransposition and integration of LINE-1 into the genome [3]. The observed radiation-induced activation of LINE-1 protein may contribute to further insertion and activation of the LINE-1 element. The active Line-1 element can and do insert into genes (very often into proto-oncogenes) thus changing their expression. Roman-Gomez and colleagues have demonstrated that epigenetic changes in the LINE-1 promoter alter the expression of c-MET oncogene that is highly expressed in CML patients [24]. c-MYC activation that depends on LINE-1 insertion has been reported in breast cancer [22]. Our results show an increased level of c-MYC protein in rat mammary tissue after radiation exposure (Fig.4, Suppl. Fig. 1). As in the LINE-1 protein, it is the highest and lowest doses of radiation that cause c-MYC protein synthesis.

In conclusion, our results present the evidence that ionizing radiation decreases CpG methylation in the LINE-1 promoter following the up-regulation of LINE-1 RNA levels and increases the synthesis of LINE-1 protein. There was no specific radiation dose-dependent response. Both low and high doses/energy levels had a similar effect on LINE-1 activation. Such LINE-1 activation may be related to the activation of the c-MYC oncogene. These findings suggest that mammary tissue exposed to genotoxic radiation may develop genomic instability due to the epigenetic activation of mobile elements and initiate cancer development.

## MATERIALS AND METHODS

### Animal models and irradiation conditions

Six-week-old intact female Long-Evans rats were obtained from Charles River (Wilmington, MA). The animals were housed two per cage in a temperature-controlled (24 °C) room in a 12-hour light-dark cycle and given *ad libitum* access to water and an NIH-31 pelleted diet. Six rats were randomly assigned to one of the following X-ray radiation treatment groups: 80kVp/0.1 Gy, 80kVp/1 Gy, 80kVp/2.5 Gy, 30kVp/0.1 Gy, and sham treated controls. Each group of animals was humanely sacrificed 6, 96 hours, and 4, 12 and 24 weeks after radiation treatment. The paired caudal inguinal mammary glands were excised. Tissue was frozen immediately in liquid nitrogen and stored at −80°C for subsequent analyses.

### Analysis of LINE-1 ORF1 methylation status by the COBRA assay

The combined bisulfite restriction analysis (COBRA) assay consisted of bisulfate modification of genomic DNA, the subsequent polymerase chain reaction (PCR) amplification and digestion of PCR product with specific restriction endonucleases [33, 45]. Genomic DNA was extracted using QiagenDNAeasy kit (Qiagen, Mississauga, Ontario, Canada) according to the manufacturer's protocol. Bisulfite conversion of genomic DNA was performed using EZ DNA Methylation-Gold Kit (Zymo Research, Irvine, CA) according to the manufacturer's protocol. Further, the bisulfite-modified DNA was PCR amplified with primers corresponding to the regulatory region of rat LINE-1 ORF1 sequence [46]. The sense primer was 5′-TTT GGT GAG TTT GGG ATA-3′ and the anti-sense primer was 5′-CTC AAA AAT ACC CAC CTA AC-3′. PCR products were digested with RsaI and BstUI restriction endonucleases (New England Biolabs, Beverly, MA) separated on 3% high resolution agarose gels (Sigma, St Louis, MO) and stained with ethidium bromide. The banding pattern analysis and the estimation of the ratio of intensities in digestion products and undigested bands was performed using both AlphaView SA 3.2.2. EXE and NIH ImageJ 1.63 Softwares.

Before conducting the above-mentioned assay, the methylation standard was made to check the untested primers. Briefly, the fully methylated and unmethylated DNA was obtained and mixed creating methylation gradient: 0%, 5%, 10%, 25%, 50%, 75% and 100% methylation. To obtain the fully methylated DNA, genomic DNA was treated with SAM and SssI methylase (NEB, Ipswich, MA). The non-methylated DNA was obtained by amplifying genomic DNA using the WGA amplification kit (Sigma St Louis, MO) according to the manufacturer's protocol. The COBRA assay was conducted on methylation gradient as described above.

### RNA isolation and quantitative real-time polymerase chain reaction (qRT-PCR)

Total RNA was isolated using the Illustra RNAspin mini kit (GE Healthcare Life Sciences, Buckinghamshire, UK). Approximately 50–70 mg of mammary gland tissue was processed following the manufacturer's instructions. Samples were eluted in Ultrapure DNase/RNase-free distilled water provided in the kit. RNA samples were quantified by ultraviolet spectroscopy (NanoDrop, Wilmington, DE).

Quantitative real-time PCR was performed to detect the expression level of LINE-1 ORF1 transcript. *β*-*Actin* was used as a reference gene. All reactions were performed using cDNA synthesized from 500 ng of RNA sample using the Bio-Rad iScript Select cDNA Synthesis Kit (Bio-Rad Laboratories, Hercules, CA). Samples were stored at −20°C for long-term storage and at 4 °C until used for the subsequent qRT-PCR reactions.

Primers were designed using the NCBI database and PrimerQuest (Integrated DNA Technologies, Inc., Coralville, IA). Primers were as follows: *LINE-1 ORF1* forward primer 5′-AAG AAA CAC CTC CCG TCA CA-3′ and reverse primer 5′-CCT CCT TAT GTT GGG CTT TAC C-3′; *beta-Actin* reference gene forward primer 5′-CCT CTG AAC CCT AAG GCC AA-3′ and reverse primer 5′-AGC CTG GAT GGC TAC GTA CA-3′. Reactions were prepared using 1 μL of diluted cDNA, 10 pmol/μL of each forward and reverse primer and SsoFast EvaGreen Supermix (Bio-Rad Laboratories, Hercules, CA) according to the manufacturer's instructions. Samples were prepared in triplicate and were run on the Bio-Rad C1000 Thermal Cycler equipped with the CFX96 Real-Time System. The qRT-PCR protocol consisted of denaturation at 95 °C for 2 min; forty-three cycles of denaturation (95 °C, 5 sec) and annealing/extension (55 °C, 5 sec); and the final extension at 65 °C for 5 sec. For every set of primers, annealing temperature optimization, melting curve analysis and gel analysis of amplicon were performed. To evaluate PCR efficiency, the standard curve was established using series of cDNA dilutions. The data were captured and organized by the Bio-Rad CFX Manager 2.1 software (Bio-Rad Laboratories, Hercules, CA).

Quantification data from the Bio-Rad CFX Manager software were analyzed in Microsoft Excel using the Pfaffl method [47]. Graphs showing a fold change from the sham group were created showing transcript regulation directions (up- or down-regulation).

### Western immunoblotting

For protein isolation, 30-50 mg of mammary gland tissue were washed in PBS, lysed, and sonicated in 0.25 mL of 1% sodium dodecyl sulphate (SDS) containing protein inhibitors. The lysates were cleared using centrifugation. The protein content was determined using the Bradford protein determination assay (BioRad, Hercules, CA). Equal amounts of lysate protein were subsequently run on 10-12% SDS-polyacrylamide gels and transferred to PVDF membranes (GE Healthcare, Baied'Urfé, Québec).

Western immunoblotting was conducted using the well-established protocols [48, 49]. The membranes were incubated with antibodies against rabbit anti-Line-1, mouse anti-c-Myc (1:100 dilution, Santa Cruz Biotechnology, Inc., Santa Cruz, CA ), and mouse anti-Actin (1:1000 dilution, Abcam Inc., Cambridge, MA). Antibody binding was revealed through the incubation with horseradish peroxidase-conjugated secondary antibodies (GE Healthcare, Piscataway, NJ) and the ECL Plus immunoblotting detection system (GE Healthcare, Piscataway, NJ). Chemiluminescence was detected using BioMax MR films (Eastman Kodak, New Haven, CT). The unaltered PVDF membranes were stained with Coomassie Blue (BioRad, Hercules, CA) to prove equal protein loading. Signals were quantified using NIH ImageJ 1.63 software and normalized to loading controls. Images are representative of two independent immunoblots. The results are presented as mean ± S.E.M. Statistical analyses were conducted using the Student's t-test. P-values less than 0.05 were considered significant.

## SUPPLEMENTARY MATERIAL FIGURE


