# Supplementary material for: Mobilization of LINE-1 in irradiated mammary gland tissue may potentially contribute to low dose radiation-induced genomic instability
Source: Genes Cancer. 2015 Jan;6(1-2):71–81. doi: 10.18632/genesandcancer.50 (PMC4362486; doi:10.18632/genesandcancer.50)
Supplement: Supplementary file 1 [file ganc-06-071-s001.pdf]

Mobilization of LINE-1 in irradiated mammary gland tissue may potentially contribute to low dose radiation-induced genomic instability

Supplementary Material

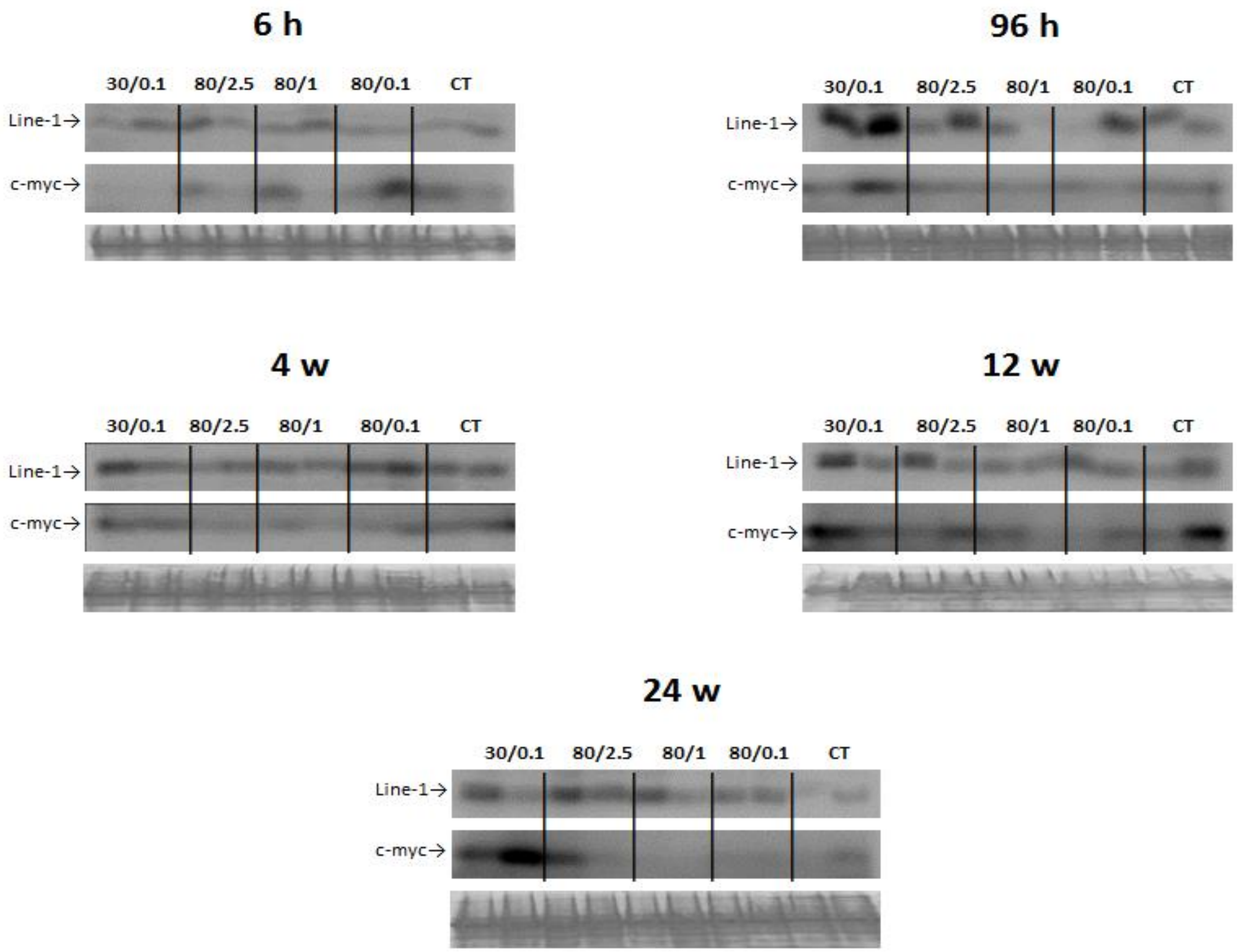

Suppl. Figure 1: LINE-1 and c-MYC levels in rat mammary gland upon whole body irradiation. Protein levels relative to those of control non-irradiated animals. Representative blots from two independent experiments.
